# V-A ECMO for neonatal coxsackievirus B fulminant myocarditis: a case report and literature review

**DOI:** 10.3389/fcvm.2024.1364289

**Published:** 2024-05-21

**Authors:** Xingchao Li, Li Sun, Shibing Xi, Yaofei Hu, Zhongqin Yu, Hui Liu, Hui Sun, Weili Jing, Li Yuan, Hongyan Liu, Tao Li

**Affiliations:** ^1^ Department of Pediatrics, Taihe Hospital, Hubei University of Medicine, Shiyan, Hubei Province, China; ^2^ Institute of Pediatric Research, Hubei University of Medicine, Shiyan, Hubei Province, China; ^3^ Institute of Pediatric Research, Taihe Hospital, Hubei University of Medicine, Shiyan, Hubei Province, China; ^4^ Department of Neonatology, Maternal and Child Health Hospital of Hubei Province, Tongji Medical College, Huazhong University of Science and Technology, Wuhan, Hubei Province, China

**Keywords:** extracorporeal membrane oxygenation (ECMO), newborn, myocarditis, enterovirus, coxsackievirus B (CVB), echocardiography, cardiac troponin I (cTnI), N-terminal pro-brain natriuretic peptide (NT-pro BNP)

## Abstract

**Background:**

Neonatal (enteroviral) myocarditis (NM/NEM) is rare but unpredictable and devastating, with high mortality and morbidity. We report a case of neonatal coxsackievirus B (CVB) fulminant myocarditis successfully treated with veno-arterial extracorporeal membrane oxygenation (V-A ECMO).

**Case presentation:**

A previously healthy 7-day-old boy presented with fever for 4 days. Progressive cardiac dysfunction (weak heart sounds, hepatomegaly, pulmonary edema, ascites, and oliguria), decreased left ventricular ejection fraction (LVEF) and fractional shortening (FS), transient ventricular fibrillation, dramatically elevated creatine kinase-MB (405.8 U/L), cardiac troponin I (25.85 ng/ml), and N-terminal pro-brain natriuretic peptide (NT-proBNP > 35,000 ng/L), and positive blood CVB ribonucleic acid indicated neonatal CVB fulminating myocarditis. It was refractory to mechanical ventilation, fluid resuscitation, inotropes, corticosteroids, intravenous immunoglobulin, and diuretics during the first 4 days of hospitalization (DOH 1–4). The deterioration was suppressed by V-A ECMO in the next 5 days (DOH 5–9), despite the occurrence of bilateral grade III intraventricular hemorrhage on DOH 7. Within the first 4 days after ECMO decannulation (DOH 10–13), he continued to improve with withdrawal of mechanical ventilation, LVEF > 60%, and FS > 30%. In the subsequent 4 days (DOH 14–17), his LVEF and FS decreased to 52% and 25%, and further dropped to 37%–38% and 17% over the next 2 days (DOH 18–19), respectively. There was no other deterioration except for cardiomegaly and paroxysmal tachypnea. Through strengthening fluid restriction and diuresis, and improving cardiopulmonary function, he restabilized. Finally, notwithstanding NT-proBNP elevation (>35,000 ng/L), cardiomegaly, and low LVEF (40%–44%) and FS (18%–21%) levels, he was discharged on DOH 26 with oral medications discontinued within 3 weeks postdischarge. In nearly three years of follow-up, he was uneventful, with interventricular septum hyperechogenic foci and mild mitral/tricuspid regurgitation.

**Conclusions:**

Dynamic cardiac function monitoring via real-time echocardiography is useful for the diagnosis and treatment of NM/NEM. As a lifesaving therapy, ECMO may improve the survival rate of patients with NM/NEM. However, the “honeymoon period” after ECMO may cause the illusion of recovery. Regardless of whether the survivors of NM/NEM have undergone ECMO, close long-term follow-up is paramount to the prompt identification and intervention of abnormalities.

## Introduction

Neonatal myocarditis (NM) is rare but unpredictable and devastating (1–6), mainly caused by enteroviruses such as coxsackievirus B (CVB) (1–9) and echovirus (7, 10). Generally, neonatal (enteroviral) myocarditis (NM/NEM) is marked by occult onset with nonspecific symptoms (1, 5, 8) and fulminant progression (1, 2, 5, 8). It is prone to cardiogenic shock, malignant arrhythmia, and multiorgan failure (2, 5, 8), with a fatality rate of 31% (4) to 38.6% (7). Although extracorporeal membrane oxygenation (ECMO) can improve the hospital survival of NM/NEM (2, 3, 5, 8, 11, 12), there are many patients with poor prognosis (11) or premature death (3, 5, 9, 11–13) unless prompt cardiac transplantation is performed (2, 13).

Neonatal ECMO is widely used for persistent pulmonary hypertension of the newborn, meconium aspiration pneumonia, congenital heart disease, congenital diaphragmatic hernia, and sometimes for myocarditis, etc (14). The success rate of ECMO in treating NM compared to neonatal respiratory diseases is low (3, 7, 11–15), which is not unrelated to the high mortality of NM itself (3, 7, 11). Here, we report a case of neonatal CVB fulminant myocarditis successfully treated with veno-arterial ECMO (V-A ECMO).

## Case presentation

A previously healthy 7-day-old boy born at 38 weeks and 2 days' gestation with a birth weight of 3.35 kg required admission due to intermittent fever for 4 days with a maximum body temperature of 38.3°C, nasal congestion, runny nose, and increasing lethargy, despite treatment with piperacillin sulbactam at the local hospital. On admission, his vital signs and body weight were axillary temperature 37.9°C, pulse rate 168 beats/min, respiratory rate 60 breaths/min, blood pressure 57/32 mmHg, and 3.65 kg, respectively. Although the heart sounds were normal, he was slightly lethargic, with a tight anterior fontanelle, stiff neck, hypertonia, dyspnea, cyanosis (SpO_2_ 75%) corrected by continuous positive airway pressure (CPAP, FiO_2_ 30%), obvious abdominal distension, weak bowel sounds, and hypoperfusion.

**Figure 1:**
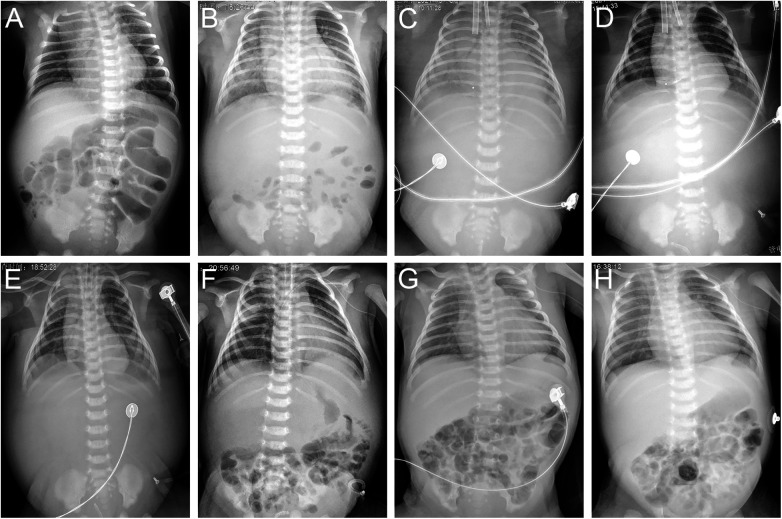
Chest and abdominal radiographs on DOH 1 (**A**), DOH 4 (**B**), DOH 6 (**C**), DOH 8 (**D**), DOH 10 (**E**), DOH 12 (**F**), DOH 19 (**G**) and DOH 21 (**H**). Increasingly intensified pulmonary edema, disappeared intestinal tube aeration and accumulated ascites (**A–C**), gradually subsided pulmonary edema, recovered intestinal tube aeration and absorbed ascites (**D–F**), enlargement of the heart shadow, relatively clear lung field and well-inflated intestinal tube (**G,H**). DOH, day of hospitalization.

**Pre-ECMO (**the first four days of hospitalization or postnatal day 7–10, **DOH 1**–**4/PND 7**–**10):** He had recurrent fever (peaked at 38.2°C), weak heart sounds (DOH 2), aggravated coma (DOH 3), obvious abdominal distension, gradual hepatomegaly to 4 cm (DOH 3), absent bowel sounds (DOH 4), systemic edema (DOH 4), exacerbation of pulmonary edema, decreased intestinal inflation, increased ascites (Figures 1A,B), oliguria (DOH 4, Table 1), transient supraventricular tachycardia and ventricular fibrillation (DOH 3), ST-segment depression in lead V1 and V3, and heart failure. His left ventricular ejection fraction (LVEF) and fractional shortening (FS) dipped as low as 40% and 18%, respectively (Table 1), with positive anal swab enterovirus ribonucleic acid (DOH 2), and markedly raised lactate (maximum value 8.4 mmol/L), creatine kinase-MB (CK-MB, maximum value 405.8 U/L), cardiac troponin I (cTnI, maximum value 25.85 ng/ml), and N-terminal pro-brain natriuretic peptide (NT-proBNP > 35,000 ng/L throughout hospitalization) (Table 1). Although timely empiric antimicrobials (meropenem 40 mg/kg IV q8h and vancomycin 10 mg/kg IV q8h), mechanical ventilation (FiO_2_ 100%), fluid resuscitation, dobutamine, dopamine, diuretics, total parenteral nutrition, intravenous immunoglobulin (685 mg/kg, qd, 3 days), epinephrine (0.2–0.5 μg/kg/min), corticosteroids, and cedilanid were performed, he continued to deteriorate.

**Table 1:** Relevant data of this newborn.

Normal range Stage		WBC	HGB	PLT	hsCRP	CK-MB	cTnI	NT-proBNP	Lactate	LVEF	FS	Fluid intake (ml/kg/d)	UV
		5–15 (10^9^/L)	150–170 (g/L)	150–350 (10^9^/L)	0–5 (mg/L)	0–24 (U/L)	0–0.3 (ng/ml)	0–300 (ng/L)	1.0–1.8 (mmol/L)	50–70	25–45		2–4 (ml/kg/h)
										(%)	(%)		
Pre-ECMO	DOH 1/PND 7	8.2	152	31	1.33	152.3	-	-	2.1	-	-	53	5.07
		7.58	140	55	1.11								
	DOH 2/PND 8	6.32	150	51	0.5	-	-	-	1.3	46	21	134	1.99
	DOH 3/PND 9	-	-	-	-	405.8	25.85	>35,000	8.4	43 (09:44)	20 (09:44)	134	1.05
										40 (17:05)	18 (17:05)		
	DOH 4/PND 10	12.13	131	87	2.8	-	-	-	4.3	53	25	93	0.62
On-ECMO	DOH 5/PND 11	20.77	114	148	1.42	126.8	9.92	>35,000	3.8	37 (10:37)	17 (10:37)	128	2.2
		9.7	131	87	0.7	58.1				35 (23:51)	16 (23:51)		
	DOH 6/PND 12	8.53	116	72	0.84	42.3	3.52	>35,000	2.2	21 (08:55)	9 (08:55)	247	13.7
										27 (17:01)	12 (17:01)		
	DOH 7/PND 13	9.45	108	168	0.5	23.6	2.56	>35,000	3.6	27 (11:00)	12 (11:00)	123	5.25
		10.5	101	193	0.5					27 (16:00)	12 (16:00)		
		8.56	115	167	-					29 (22:00)	12 (22:00)		
	DOH 8/PND 14	8.06	119	159	0.6	29.2	3.8	>35,000	1.6	**30** **(10:58)**	**13** **(10:58)**	123	4.11
		7.42	106	142	0.5					**37** **(17:36)**	**16** **(17:36)**		
	DOH 9/PND 15	7.32	103	133	0.64	24.3	1.96	>35,000	3.5	**34** **(10:44)**	**15** **(10:44)**	122	4.77
		10.9	120	143	0.5					54 (16:50)	-		
Post-ECMO (early stage)	DOH 10/PND 16	9.39	120	131	0.5	8.3	1.89	>35,000	1.5	66 (12:35)	33 (12:35)	113	3.3
		15.54	126	143	0.5					64 (15:59)	32 (15:59)		
	DOH 11/PND 17	13.07	111	98	0.6	15.6	0.7	>35,000	1.8	62	30	101	2.3
	DOH 12/PND 18	9.71	95	108	0.5	30	0.33	>35,000	1.8	62	31	104	4.4
	DOH 13/PND 19	8.58	97	135	1.1	26.7	0.27	>35,000	1.2	62	32	99	2.16
Post-ECMO (middle stage)	DOH 14/PND 20	9.35	138	182	1.0	-	-	-	2.6	57	28	118	2.16
	DOH 15/PND 21	9.69	139	213	0.51	-	-	-	2.2	-	-	107	2.1
	DOH 16/PND 22	-	-	-	-	-	-	-	-	-	-	106	2.5
	DOH 17/PND 23	8.77	114	168	1.9	11.3	0.13	>35,000	1.2	52	25	91	1.48
Post-ECMO (later stage)	DOH 18/PND 24	6.88	111	156	4.5	-	-	-	1.3	46 (12:15)	22 (12:15)	91	3.9
										39 (16:46)	18 (16:46)		
	DOH 19/PND 25	7.53	125	192	2.5	27.1	0.28	>35,000	2.9	37 (11:27)	17 (11:27)	115	4.6
										38 (17:28)	17 (17:28)		
Post-ECMO (stable stage)	DOH 20/PND 26	-	-	-	-	-	-	-	-	43 (10:00)	20 (10:00)	104–120	2.0–3.3
										42 (16:44)	20 (16:44)		
	DOH 21/PND 27	-	-	-	-	-	-	-	-	44	21		
	DOH 22/PND 28	-	-	-	-	-	-	-	-	43	20		
	DOH 23/PND 29	-	-	-	-	-	-	-	-	41	18		
	DOH 24/PND 30	5.67	109	184	1.39	24.6	0.32	>35,000	-	41	19		
	DOH 25/PND 31	-	-	-	-	-	-	-	-	41	19		
	DOH 26/PND 32	-	-	-	-	-	-	-	-	40	18		
Follow-up	PND 41	5.92	121	345	0.5	59.2	-	-	-	49	23	-	-
	PND 56	-	-	-	-	-	-	-	-	66	34		
	PND 70	-	-	-	-	25.2	0.07	760.8	-	-	-		
	PND 85	-	-	-	-	-	-	-	-	66	34		
	PND 121	-	-	-	-	-	-	-	-	NA	NA		
	PND 205	-	-	-	-	-	-	-	-	NA	NA		
	PND 498	-	-	-	-	-	-	-	-	67	36		
	PND 1,084	-	-	-	-	-	-	-	-	60	31		

WBC, white blood cell; HGB, hemoglobin; PLT, platelet; hsCRP, hypersensitive C-reactive protein; CK-MB, creatine kinase-MB; cTnI, cardiac troponin I; NT-proBNP, N-terminal pro-brain natriuretic peptide; LVEF, left ventricular ejection fraction; FS, fractional shortening; UV, urine volume; ECMO, extracorporeal membrane oxygenation; DOH, day of hospitalization; PND, postnatal day; NA, not available. The bolded LVEF and FS values were measured at a temporary ECMO flow of 0.1 L/min.

**On-ECMO (DOH 5–9/PND 11–15):** Heart failure, cardiogenic shock, hypoperfusion, hyperlactatemia, and oliguria were unresponsive to the above conventional therapies. Furthermore, his LVEF, FS, and urine output further decreased to 37% (DOH 5 10:37, Table 1), 17% (DOH 5 10:37, Table 1), and 25 ml (DOH 5 08:00-21:00), respectively. V-A ECMO was initiated at 22:00 on DOH 5 via the right internal jugular vein (12 Fr venous cannula) and common carotid artery (8 Fr arterial cannula) with a pump flow of 0.45 L/min. After ECMO support (pump flow 0.4–0.45 L/min) with continuous sedation (midazolam), analgesia (sufentanil), and muscle relaxation (vecuronium bromide), he was no longer feverish with renormalization of urine volume (Table 1) and significant reduction of systemic edema (DOH 9) and pulmonary edema (Figures 1C,D), and abdominal bloating subsided on DOH 9, though there were weak heart sounds, a 3 cm hepatomegaly, no bowel sounds and intestinal inflation, persistent ascites despite timely abdominal drainage via vein detained needle (24 G) on the afternoon of DOH 6 without complications, and bilateral renal enlargement detected by point-of-care ultrasonography (POCUS) (DOH 5). The lowest levels of lactate, CK-MB, and cTnI were 1.6 mmol/L, 23.6 U/L, and 1.96 ng/ml, respectively (Table 1). His blood CVB ribonucleic acid was positive on DOH 9. SvO_2_, activated partial thromboplastin time (APTT, intravenous heparin 2.5–35 units/kg/h), mean arterial pressure (MAP, intravenous sodium nitroprusside 0.5–7 µg/kg/min), and hemoglobin were maintained at 73.6–80.9% (target value 65%–80%), 62–120 s (target value 60–80 s), 37–82 mmHg (target value 40–50 mmHg), and 101–120 g/L (target value 120–140 g/L), respectively. Although symptoms of heart failure were relieved, LVEF and FS remained at low levels of 21%–54% and 9%–17%, respectively (Table 1). Bilateral grade III intraventricular hemorrhage (IVH) was detected at DOH 7 by daily POCUS, and kept static thereafter through strengthening liquid restriction (from 247 ml/kg/d to 122–123 ml/kg/d, Table 1), decreasing heparin dosage (from 35 units/kg/h to 20–25 units/kg/h) to limit APTT mainly between 66 and 100 s, downregulating ECMO flow (from 0.45 L/min to 0.4 L/min) and upregulating sodium nitroprusside dosage (from 2.25 µg/kg/min to 7 µg/kg/min) to limit MAP mainly between 40 and 60 mmHg, and plasma infusion (35 ml on DOH 7, 50 ml on DOH 8). We attempted to dynamically assess the patient's cardiac function such as LVEF by temporarily reducing the ECMO flow to 0.1–0.15 L/min on the morning and afternoon of DOH 8 and DOH 9, which was 30% (Table 1, ECMO flow 0.1 L/min), 37% (Table 1, ECMO flow 0.1 L/min), 34% (Table 1, ECMO flow 0.1 L/min), and 70% (ECMO flow 0.15 L/min), respectively.

**Post-ECMO (early stage) (DOH 10–13/PND 16–19):** Owing to the improvement under V-A ECMO support, we gradually lowered the ECMO flow from 0.4 L/min to 0.1 L/min since DOH 10 08:30 by 0.05 L/min every half hour in an attempt to withdraw ECMO. The LVEF measured at 09:30 on DOH 10 was 52% (ECMO flow 0.3 L/min). Finally, he was weaned from ECMO at 12:00 on DOH 10, without clinical deterioration. After weaning from ECMO, his breathing remained stable from synchronized intermittent mandatory ventilation (SIMV) to nasal intermittent positive pressure ventilation (NIPPV). Soft tissue edema (DOH 11), pulmonary edema (Figures 1E,F), and ascites (Figures 1E,F) gradually subsided. His consciousness, heart sounds (DOH 11), bowel sounds (DOH 13), liver (DOH 13), kidneys (DOH 12), intestinal inflation (Figures 1E,F), and electrocardiogram were restored, and bilateral IVH did not progress. Lactate, CK-MB, and cTnI nearly normalized (Table 1). Continuous low-dose intravenous epinephrine (gradually decreased from 0.1 to 0.03 µg/kg/min) and diuretics can maintain LVEF > 60% and FS > 30% (Table 1).

**Post-ECMO (middle stage) (DOH 14–17/PND 20–23):** After discontinuing epinephrine and diuretics, LVEF and FS decreased to 52% and 25%, respectively (Table 1), and his heart except for the right ventricle began to enlarge on DOH 17, but other than that the overall clinical improvement was manifested by fine tolerance after formula feeding (DOH 15), stable breathing under noninvasive ventilation support (NIPPV→CPAP→HFNC, high flow nasal cannula), normal cTnI (Table 1) and electrocardiogram, and absorption of bilateral IVH (DOH 17).

**Post-ECMO (later stage) (DOH 18–19/PND 24–25):** Although NT-proBNP kept above 35,000 ng/L, LVEF and FS further dropped to 37%–38% and 17%, respectively (Table 1), and four chambers of the heart enlarged (DOH 18), except for paroxysmal mild tachypnea eliminated by timely strengthening fluid restriction and diuresis, and reuse of cedilanid and CPAP, there was no other clinical deterioration with clear lung fields and good intestinal inflation (DOH 19, Figure 1G).

**Post-ECMO (stable stage) (DOH 20–26/PND 26–32):** Respiratory stabilization continued with reduced support (CPAP→HFNC→room air). Despite NT-proBNP elevation (> 35,000 ng/L), cardiomegaly, and low LVEF (40%–44%) and FS (18%–21%) levels, he gradually stabilized and was successfully transitioned to an oral regimen of digoxin, captopril, spironolactone, and prednisolone at DOH 20. X-ray image (DOH 21, Figure 1H) showed no significant change compared to Figure 1G. Finally, the patient was discharged on DOH 26.

**Figure 2:**
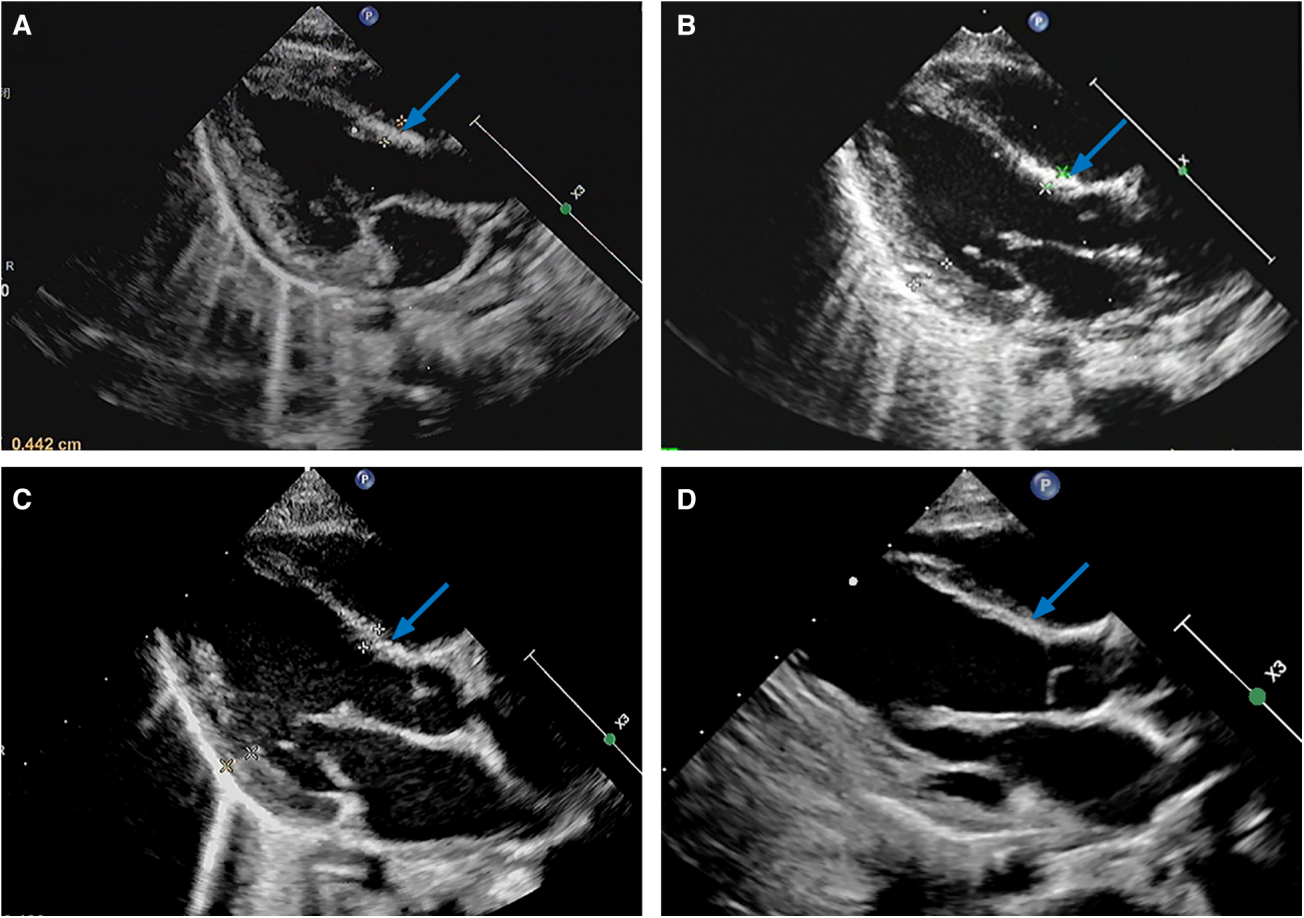
Parasternal left ventricle long axis view of transthoracic echocardiograms on PND 41 (**A**), PND 85 (**B**), PND 498 (**C**), and PND 1,084 (**D**), illustrating diffuse hyperechogenic foci of the interventricular septum (arrows). PND, postnatal day.

**Follow-up (PND 33**-**):** Within 3 weeks postdischarge, all the oral medications were discontinued. To date, in nearly three years of follow-up, LVEF (PND 56, Table 1), FS (PND 56, Table 1), and heart size (PND 85) gradually normalized, and NT-proBNP decreased to 760.8 ng/L (PND 70, Table 1). There were no other abnormalities aside from interventricular septum hyperechogenic foci (Figure 2) and mild mitral/tricuspid regurgitation, and the former had become less pronounced by PND 1,084 (Figure 2D).

## Discussion

Enteroviruses, represented by CVB and echovirus (16, 17), can cause both neonatal community-acquired and hospital-associated infections (17, 18, 19). The former is sporadic with an epidemic season of summer and fall (4, 18), while the latter is prone to outbreak in the neonatal room (19), and both may be fatal (18, 20). As our case occurred in later spring, it is equally important to timely identify and treat sporadic (severe) cases in the nonepidemic season.

Newborns are susceptible to CVB (4), the commonest pathogen causing NM (3, 4, 7). Usually, neonatal CVB infection is occult-onset with such nonspecific or sepsis-like symptoms as fever or hypothermia (3, 4, 7, 8, 17, 18), poor feeding (3–5, 7, 8, 16, 18), lethargy (3, 4, 7, 8, 16, 17), diarrhea (3, 17), apnea or tachypnea (3–5, 17, 18), seizure (3, 18), irritability (7, 17), hypotonia (7), hypoperfusion (4, 7, 17), jaundice (16–18), and hepatomegaly or hepatosplenomegaly (4, 16). In addition, abdominal distension caused by CVB infectious-toxic enteroplegia, and respiratory catarrhal symptoms were present in our case, along with transient leukocytosis, initial thrombocytopenia, and consistently normal hypersensitive C-reactive protein (Table 1). Neonatal enterovirus infection (including CVB) mostly remains asymptomatic (5, 13), and the majority of symptomatic neonates are self-limited or mild (5, 17). Only a few cases rapidly deteriorate into multiorgan damage or failure (e.g., myocardium, central nervous system, liver, and coagulation) (7, 16, 17, 21) through direct injury (6), inflammatory infiltration (6), and/or immune injury (22). If so, it is more severe than that in older children (17). Severe neonatal enterovirus infection is more likely to occur in preterm and male infants, neonates with onset < PND 7, and those infected with highly virulent strains (CVB 1–5, echovirus 5, 6, 7, 9, 11, 14, 17, 19, 21) and higher blood viral loads (17), while neonatal CVB myocarditis usually occurs in male infants (4, 9) younger than PND 14 (4, 9, 18), as shown in our case.

Unlike pediatric myocarditis, NM/NEM is more prone to rapid progression to fulminant cardiovascular collapse (23), with a high risk of early mortality and late dysfunction (3, 4, 7, 13). According to previous researches, the survival rate of NEM patients supported with ECMO is only 52% (15), 42.9% (3), 39.5% (7), or even as low as 33% (12). NEM can develop sequelae such as myocardial calcification (2, 24), left ventricular aneurysm (LVA) (4, 13), and dilated cardiomyopathy (DCM) (4, 24). These may correlate with immature neonatal immune (7, 18) and myocardial (25) development, lack of maternal neutralizing antibody (18), insufficient neonatal cardiac compensatory capacity, and poor neonatal cardiac ability to tolerate injury and to postinjury repair. Normally, neonatal CVB infection can be rapidly confirmed by PCR (26). In this case report, we focus on the following issues.

### “Refractory” ascites

Systemic edema in this case was caused by heart failure and capillary leak secondary to CVB infection, and reached its peak along with fluid overload at ECMO initiation (14). Subsequently, owing to the efficacy of ECMO, soft tissue edema and pulmonary edema were gradually alleviated. However, despite timely abdominal drainage, ascites persisted (Figure 1D). This may be associated with the relative insufficiency of gastrointestinal blood flow because of the unrestored gastrointestinal peristalsis caused by infectious toxic enteroplegia. Moreover, the usage of sedatives, analgesics, and muscle relaxants during ECMO further inhibited gastrointestinal motility and blood supply, which jointly limited the absorption of ascites by the peritoneal capillary network, and postural restriction during ECMO additionally hindered the discharge of ascites through the abdominal drainage tube. With the cessation of ECMO, the lifting of postural restriction, the reduction of sedatives, analgesic, and muscle relaxants, and the recovery of gastrointestinal function, yellow ascites was soon discharged through the abdominal drainage tube by changing posture and no longer recurred. Therefore, during the treatment of NM/NEM with ECMO, if the gastrointestinal tract is not inflated and/or ascites is persistent, it should not be pessimistically assumed that the child has necrotizing enterocolitis induced by enterovirus infection unless there are clear evidences.

### Static IVH

Neonatal IVH often occurs in preterm infants rather than term infants, and its most important pathogenesis is ascribed to the fragility of germinal matrix vasculature, and the anomalies in cerebral blood flow, platelet, and coagulation (27). Severe intracranial hemorrhage, the most serious complication of neonatal ECMO, may not only lead to premature ECMO decannulation but also be life-threatening (14). In our case, bilateral grade III IVH occurred on the third ECMO day, closely related to anticoagulation and fluctuations in blood volume and pressure. Meanwhile, fluid overload at ECMO initiation may have inestimable impacts on cerebral blood perfusion. Inevitable iatrogenic blockage of the right internal jugular vein and common carotid artery blood flow due to ECMO cannulation may also be involved. When this case of IVH occurred, the newborn had not yet met the decannulation criteria, while blind decannulation may cause all in vain. Unless absolutely necessary, the newborn should not be weaned from ECMO. At this moment, it is necessary to further optimize the cardiovascular and coagulation status and to closely monitor the changes in IVH via POCUS (14). If intracranial hemorrhage develops rapidly, ECMO must be terminated (14). Fortunately, the IVH in our case remained static and was finally absorbed.

### Relapse after the “honeymoon period”

His heart had got rest on V-A ECMO, so that in the first 4 days post-ECMO his condition continued to improve, and LVEF and FS were maintained above 60% and 30%, respectively. He perfectly passed the “honeymoon period”, causing a “false recovery”. Thereafter, because of incomplete recovery, his heart was overwhelmed again, inducing decline of LVEF and FS, tachypnea, and cardiomegaly. After strengthening fluid restriction and diuresis and improving cardiopulmonary function, he was discharged smoothly. However, it should be noted that in the second stage of myocarditis pathophysiology (within several weeks after presentation), the body may suffer acquired T-cell and B-cell immune responses, leading to persistent myocardial damage or even cardiac dysfunction (28). In our case, the relapse of heart failure post-ECMO tends to be caused by incomplete cardiac recovery rather than acquired immune responses mediated myocardial damage.

### Unpredictability

In neonates, persistently increased CK-MB, cTnI, and NT-proBNP, together with significantly reduced LVEF and FS commonly indicate NM after excluding asphyxia, etc., particularly when there is an enterovirus infection prodrome. Through our case, we believe that combined monitoring of the dynamic changes in heart sounds, rhythm, cardiac function, CK-MB, cTnI, NT-proBNP, LVEF, and FS is useful for the diagnosis of NM/NEM, and that the levels of NT-proBNP, LVEF, and FS can also reflect whether the child is recovered to some extent. However, how to early diagnose NM/NEM and how to accurately predict its development trend are more important for prompt precision therapy and improvement of the outcome. The development of more perfect biomarkers and monitoring methods for myocardial injury is expected to enable early diagnosis and intervention of NM/NEM, thereby ameliorating its prognosis.

Compared to myoglobin, heart type fatty-acid-binding protein, with earlier release and higher cardiac specificity, is an ideal biomarker for the early detection of myocardial injury (29). It can increase within 1.5 h after myocardial injury, peak at approximately 6 h, and return to baseline concentration in 24 h (29). However, myoglobin rises within 1–3 h after myocardial injury, peaks at 6–9 h, and returns to normal range in 24 h (30), and the variation patterns of cTnI and CK-MB are 3–4 h/16–18 h/2 w (31) and 4–6 h/16–18 h/48–72 h (32), respectively.

Cardiac magnetic resonance (CMR) imaging, compared with echocardiography, may have advantage in the early diagnosis and late follow-up of both pediatric and adult myocarditis (22, 33). The 2018 Lake Louise Criteria improves CMR accuracy in diagnosing (acute) myocardial inflammation (34). CMR is beneficial for the detection of LVEF-preserved myocarditis and suspected myocarditis (33), but it requires sophisticated techniques (22). Moreover, upon suspicion of NM/NEM, the critically ill neonates may require intensive cardiopulmonary support and may deteriorate any minute, so pursuing CMR scan at this moment is risky, not to mention its time-consuming nature. Howbeit, whenever this happens, the real-time and portability of POCUS will be highlighted (22).

Notably, increased wall thickness suggesting myocardial inflammatory edema may precede cardiomegaly in myocarditis (35), which can be detected by initial echocardiography. Compared to conventional echocardiography, speckle tracking echocardiography can detect myocardial dysfunction early (36) even in patients with LVEF-preserved myocarditis (37), is highly consistent with CMR in the diagnostic and prognostic evaluation of myocarditis (38), and is helpful in identifying pediatric myocarditis at risk for arrhythmias (39). Furthermore, in NEM, compared to newborns who died or needed cardiac transplant, transplant-free survivors have better global longitudinal and circumferential strain at the initial echocardiography without differences in demographic or laboratory variables (age at onset, sex, gestational age, birth weight, cTnI, creatine kinase, BNP, or NT-proBNP) and in other echocardiographic measures (LVEF and LV diastolic/systolic diameter/volume), though the latter may ultimately live with common sequelae such as chronic LVA and left ventricular dysfunction (13).

Although ECMO may improve the hospital survival of NM/NEM patients, there is no large multicenter research to decipher the long-term prognosis of NM/NEM patients supported with ECMO (3). NM/NEM may develop sequelae such as interventricular septum hyperechogenic foci (2, 24), LVA (4, 13), and DCM (4, 24, 33). The pathogenesis of LVA secondary to viral myocarditis is unclear (13), which may be in connection with the long-term fiber scar repair of the necrotic myocardium (4), while Th17 cells/IL-17A-mediated autoimmunity and myocardial fibrosis may involve DCM formation post-myocarditis (40). Nevertheless, the mechanism determining the chronic transformation of cardiac dysfunction post-myocarditis and the reason why some patients cannot recover have not yet been fully elucidated (33), which may be linked to the persistence of viral infection, inflammatory damage, and immune reaction (22, 28). Additionally, genetic factors such as deleterious variants involving myocardial structure and function genes might play an important role in the phenotypic outcome of myocarditis (33, 35). Currently, there are interventricular septum hyperechogenic foci without significant expansion left in our case (Figure 2), which have proven to be dystrophic calcifications after myocardial necrosis according to previous studies (2, 24). LVA and DCM usually occur within weeks to months (4) and months to years (4, 33) post-myocarditis, respectively, so the probability of LVA and DCM may gradually decrease over time in our case.

Taken together, in this case of NM/NEM, we closely monitored the changes in neonatal condition through POCUS, etc., ensured the normal operation of ECMO and efficacy, maximized the prevention and treatment of the concomitant complications of ECMO, and the newborn eventually recovered smoothly. However, limited to sample size, the following conclusions should be cautiously extrapolated.

## Conclusions and prospects

Although NM/NEM is rare, it is unpredictable with occult onset, fulminant processes, high mortality, and poor prognosis. Dynamic cardiac function monitoring via POCUS is of great value in the diagnosis and treatment of NM/NEM. As a lifesaving option, ECMO may improve the survival rate of patients with NM/NEM. However, the management of the peri-ECMO period is extremely important, especially during the “honeymoon period” after ECMO, which may easily cause the illusion of recovery. Regardless of whether the survivors of NM/NEM have undergone ECMO, close long-term follow-up is crucial to the early identification and timely intervention of abnormalities to improve their quality of life. The most difficult to predict may be that which newborns infected with enteroviruses will experience NM/NEM. More researches are imperative to further detailed, reliable, and feasible prevention and control consensus for NM/NEM.

## Data Availability

The original contributions presented in the study are included in the article/Supplementary Material, further inquiries can be directed to the corresponding authors.
